# Efficacy of Antimicrobial Catheters for Prevention of Catheter-Associated Urinary Tract Infection in Acute Cerebral Infarction

**DOI:** 10.2188/jea.JE20170022

**Published:** 2018-01-05

**Authors:** Keiji Muramatsu, Yoshihisa Fujino, Tatsuhiko Kubo, Makoto Otani, Kiyohide Fushimi, Shinya Matsuda

**Affiliations:** 1 Department of Preventive Medicine and Community Health, School of Medicine, University of Occupational and Environmental Health, Japan; 2 Data Science Center of Occupational Health, University of Occupational and Environmental Health, Japan; 3 Department of Health Policy and Informatics, Tokyo Medical and Dental University Graduate School, Japan

**Keywords:** catheter-associated urinary tract infection, antimicrobial catheter, cerebral infarction, diagnosis procedure combination, diabetes

## Abstract

**Background:**

Catheter-associated urinary tract infection (CAUTI) is a common nosocomial infection. However, the effectiveness of antimicrobial catheters in reducing CAUTI in cerebral infarction patients is unknown. The purpose of this study was to determine whether antimicrobial catheters protect against CAUTI in cerebral infarction patients.

**Methods:**

We identified 27,548 patients from the Japanese Diagnosis Procedure Combination Database who had been admitted from April 1, 2012 through March 31, 2014 for acute management of cerebral infarction and had used at least an indwelling urethral catheter. We extracted data on patient sex, age, comorbidity, length of stay, activities of daily living (ADL), surgery, hospital case volume, and catheter type. We defined CAUTI as a urinary tract infection arising during admission. We performed multi-level logistic regression analysis to analyze the reduction in CAUTI using antimicrobial catheters.

**Results:**

The rate of CAUTI was 8.8% and 8.3% in the control and antimicrobial catheter groups, respectively. Significant risk factors for CAUTI were age, diabetes requiring insulin therapy, low ADL score, and long hospitalization. Incidence rate was significantly lower in operated cases and those treated with tissue plasminogen activator. For all cases overall, the use of an antimicrobial catheter was not associated with a lower CAUTI rate. However, use was associated with a lower rate of CAUTI in diabetic patients on insulin.

**Conclusions:**

Antimicrobial catheter use was not associated with a lower incidence rate of CAUTI in acute cerebral infarction patients. However, stratified analysis suggested that use was associated with a lower incidence in diabetic patients on insulin.

## INTRODUCTION

Catheter-associated urinary tract infection (CAUTI) are common healthcare-associated infections. The National Healthcare Safety Network in the United States reported that 42,562 CAUTI occurred in 4,567 participating American hospitals in 2013.^1^ CAUTI increase medical costs and prolong hospitalization. A meta-analysis showed that CAUTI cost about US$900–2012 per case,^2^ while a prospective cohort study reported that CAUTI prolonged the hospital stay of intensive care unit patients by 10 extra days.^3^ Known measures to prevent CAUTI are catheter insertion in the operating room or another clean environment, training for catheter insertion and early catheter removal.^4^^–^^6^ Antimicrobial indwelling urethral catheters mixed or coated with antibacterial agents, including silver hydrogel and nitrofural, are considered effective in preventing CAUTI because of suppression of bacterial growth on the catheter surface.^7^

Although previous studies have shown the effectiveness of antimicrobial catheters in preventing CAUTI, the evidence is not clear. A multicenter randomized study showed no significant difference in the incidence rates of CAUTI between the silver alloy catheter group and polytetrafluoroethylene (PTFE) control group (12.5% vs 12.6%, *P* = 0.69).^8^ In that study, the nitrofural catheter group had a significantly lower incidence rate than the PTFE control group (10.6% vs 12.6%, *P* = 0.031). Another multicenter study reported that the silver alloy hydrogel catheter group had a significantly lower incidence rate than the standard catheter group (0.25/1000 patient days vs 0.60/1000 patient days, *P* < 0.001).^9^ In its Guidelines for Prevention of CAUTI, the Centers for Disease Control and Prevention recommended additional research to verify the effectiveness of antimicrobial catheters.^6^

Cerebral infarction is considered to increase the incidence of CAUTI. Cerebral infarction is a common neurological disorder and is strongly associated with urinary tract infection occurring as a result of bladder dysfunction.^6^^,^^10^^–^^12^ A prospective study reported that urinary tract infections were likely to complicate cerebral infarction and were associated with poor outcomes.^13^ Despite the susceptibility of patients with cerebral infarction to CAUTI, evidence for the efficacy of antimicrobial catheters in preventing CAUTI in these patients is lacking.

Here, we examined the effect of antimicrobial catheters in preventing CAUTI in patients with cerebral infarction using nationally administered health claims data from the Diagnosis Procedure Combination database, the provider reimbursement system for the Japanese national healthcare insurance.

## METHODS

### Study design

The study was conducted under a cross-sectional design using the Japanese Diagnosis Procedure Combination (DPC) inpatient database for the period April 2012 through March 2014. Data were collected by the DPC research group, which is funded by the Ministry of Health, Labour, and Welfare, Japan. In the study period, 1,181 hospitals participated in the survey of DPC research group and provided their DPC data to the database for research purpose. This database contains patient information and detailed procedures for the Japanese national insurance system.^14^ The research protocol was approved by the ethics committee of medical care and research of Tokyo Medical and Dental University, Japan.

### Inclusion and exclusion criteria

We selected patients whose principal diagnosis was cerebral infarction (ICD-10 code beginning with I63; *n* = 233,661). Given the homogeneous clinical course of patients, we selected those patients who were admitted to hospital within 3 days from stroke onset and who were treated with edaravone or tissue plasminogen activator (*n* = 158,089), which are commonly used in acute stroke therapy in Japan. Next, we further selected for patients who underwent insertion of an indwelling urethral catheter at least once during hospitalization (*n* = 46,074). We excluded cases in which antibiotics were administered in the first 2 days of hospitalization (*n* = 6,497) and those with complicating aspiration pneumonia (ICD-10 code beginning with J69; *n* = 6,339) during hospitalization because a previous study reported that the administration of antibiotics reduced the incidence rate of CAUTI. We also excluded patients who died within 24 hours after admission (*n* = 162) and those whose activities of daily living (ADL) score at admission was not available (*n* = 8,981).

### Type of indwelling urethral catheter and other covariates

We defined antimicrobial catheters as double-lumen catheters coated with antimicrobial agents, made with antimicrobial-mixed latex or antimicrobial-mixed silicon. We excluded patients who used both types during the course of hospitalization (*n* = 1,482).

Sex, age, cancer, diabetes, length of stay, and surgery were considered as risk factors for CAUTI, as per previous reports.^6^ Administration of tissue plasminogen activator was used as a variable to distinguish patients who received early treatment. We divided diabetic patients according to the use of insulin during the hospital stay. The severity of cerebral infarction at admission was graded using ADL score on a 20-point scale. We classified ADL performance into the four categories of very good (over 15), good (10 to 14), poor (5 to 9), and very poor (4 or less). We used the number of cases during the study period as the hospital factor. We defined hospital case volume as the number of cerebral infarction patients who had entered the hospital within 3 days from stroke onset and had been treated with edaravone or tissue plasminogen activator and had an indwelling urethral catheter inserted at least once in each hospital in the study period. These were categorized into tertile groups of 1–14 patients, 15–49 patients, and over 50 patients.

### Outcome variables

Patients were considered to have complications of CAUTI if they were diagnosed after admission with acute tubulointerstitial nephritis (ICD-10 code, N10); pyonephrosis (ICD-10 code, N13.6); renal and perinephric abscess (ICD-10 code, N15.1); urinary tract infection, site not specified (ICD-10 code, N39.0); or infection and inflammatory reaction due to prosthetic device, implant, or graft in the urinary system (ICD-10 code, T83.5).

### Statistical analyses

The association of antimicrobial catheter use with CAUTI incidence was estimated using multilevel logistic regression models, with a two-level structure of individuals nested within the 985 hospitals. We conducted additional analyses that stratified patients at risk of CAUTI. All statistical analyses were conducted using Stata version 14 (Stata Corp, College Station, TX, USA).

## RESULTS

Table 1 shows patient characteristics and CAUTI incidence rate. The rate of antimicrobial catheter use was 43%. There were no apparent differences between the standard catheter group and antimicrobial catheter group in any characteristic except surgery (16% vs 20%).

**Table 1.** Characteristics of patients and catheter-associated urinary tract infection incidence rate

	Standard catheter (*n* = 15 627)		Antimicrobial catheter (*n* = 11 921)	
	
	number of subjects or mean	% or SD	number of subjects or mean	% or SD
**Patient factor**				
Sex, female	8,223	52	6,257	52
Age, years, mean (SD)	76	(12)	76	(12)
Comorbidity				
Cancer, yes	957	6.1	779	6.5
Diabetes				
Without diabetes	12,003	77	9,204	77
With diabetes, not on insulin	1,673	11	1,186	10
With diabetes, on insulin	1,951	12	1,531	13

Length of stay, days, mean (SD)	41	(45)	41	(41)
ADL				
Very good	1,621	10	1,318	11
Good	657	4.2	532	4.5
Poor	1,559	10	1,203	10
Very poor	11,790	75	8,868	74
Had surgery	2,549	16	2,369	20
Had tPA	2,768	18	1,941	16

**Hospital factor**				
Number of cases during study period				
1–14	647	4.1	459	3.9
15–49	3,454	22	2,353	20
≥50	11,526	74	9,109	76

CAUTI, yes	1,378	8.8	994	8.3
CAUTI, per 1000 patient days	2.2		2.1	

CAUTI, catheter-associated urinary tract infection; ADL, activities of daily living; tPA, tissue plasminogen activator; SD, standard deviation.

The results of multi-level logistic regression analysis are presented in Table 2. Use of an antimicrobial catheter was not significantly associated with incidence rate of CAUTI compared with use of a standard catheter in univariate analysis (OR 0.90; 95% CI, 0.78–1.03). In the multivariate, multi-level logistic regression analysis, no significant difference in the incidence rate of CAUTI was seen between the standard catheter group and antimicrobial catheter group (OR 0.92; 95% CI, 0.80–1.07). Factors associated with a significantly increased rate of CAUTI were age, cancer, diabetes treated with insulin, poor or very poor ADL score, length of stay, and hospital case volume. Surgery and use of tissue plasminogen activator were significantly associated with a lower incidence rate of CAUTI.

**Table 2.** Odds ratios for catheter-associated urinary tract infection by multi-level logistic regression

	Univariate			Multivariate		
	
	OR	95% CI	*P*	OR	95% CI	*P*
Use of antimicrobial catheter	0.9	0.78–1.03	0.13	0.92	0.80–1.07	0.28
Female	1.32	1.21–1.44	<0.001	1.07	0.98–1.18	0.14
Age	1.03	1.03–1.04	<0.001	1.03	1.03–1.03	<0.001
Cancer	1.18	0.99–1.40	0.058	1.19	1.00–1.42	0.045
Diabetes (vs without diabetes)						
With diabetes, not on insulin	0.82	0.70–0.96	0.012	0.91	0.78–1.07	0.24
With diabetes, on insulin	1.35	1.19–1.52		1.43	1.26–1.63	<0.001
ADL (vs very good)						
Good	1.24	0.91–1.68	0.17	1.14	0.84–1.55	0.41
Poor	1.49	1.18–1.89	0.001	1.33	1.05–1.69	0.019
Very poor	2.06	1.71–2.49	<0.001	1.67	1.37–2.02	<0.001
Length of stay	1.01	1.01–1.01	<0.001	1.01	1.01–1.01	<0.001
Underwent surgery	0.92	0.82–1.04	0.21	0.81	0.71–0.92	0.001
Received tPA	0.68	0.60–0.78	<0.001	0.72	0.63–0.83	<0.001
Number of cases during study period (vs 1–14)						
15–49	0.96	0.71–1.30	0.773	1.21	0.89–1.65	0.222
≥50	1.22	0.92–1.63	0.174	1.63	1.22–2.20	0.001

ADL, activities of daily living; tPA, tissue plasminogen activator; OR, odds ratio.

Table 3 shows a stratified analysis based on age, cancer, diabetes, ADL performance, number of days of hospitalization, surgery, and use of tissue plasminogen activator. Use of antimicrobial catheters was significantly associated with a lower CAUTI incidence rate in the group of diabetics who used insulin during hospitalization; specifically, diabetic patients treated with insulin in the antimicrobial catheter group had a significantly lower incidence rate of CAUTI compared with those in the standard catheter group (OR 0.68; 95% CI, 0.52–0.89).

**Table 3.** Stratified analyses of antimicrobial catheters in the prevention of catheter-associated urinary tract infection

Conditions of stratification	OR of antimicrobial catheter	95% CI	*P*
Age, years			
≤64	0.93	0.65–1.32	0.67
65–74	0.97	0.76–1.25	0.82
75–84	0.94	0.76–1.15	0.54
≥85	0.99	0.8–1.21	0.89

Cancer			
without cancer	0.92	0.79–1.06	0.25
with cancer	1.02	0.72–1.46	0.91

Diabetes			
without diabetes	1.01	0.86–1.18	0.95
with diabetes	0.78	0.62–0.98	0.03
not on insulin	1.04	0.73–1.47	0.85
on insulin	0.68	0.52–0.89	0.005

ADL			
Very good	0.93	0.6–1.45	0.74
Good	0.75	0.44–1.28	0.29
Poor	0.85	0.6–1.2	0.36
Very poor	0.95	0.81–1.11	0.50

Length of stay			
Less than 15 days	1.12	0.59–2.12	0.73
Less than 30 days	0.90	0.71–1.15	0.42
Less than 60 days	0.91	0.76–1.1	0.35
Less than 90 days	1.17	0.86–1.6	0.32
Over 90 days	0.83	0.59–1.18	0.30

Surgery			
Underwent surgery	0.93	0.8–1.09	0.37
No surgery	0.96	0.75–1.24	0.75

Tissue plasminogen activator			
Administered tPA	0.90	0.77–1.04	0.16
No tPA	1.24	0.91–1.7	0.18

CAUTI, catheter-associated urinary tract infection; ADL, activities of daily living; tPA, tissue plasminogen activator; OR, odds ratio.

## DISCUSSION

This study investigated the association between the use of antimicrobial catheters and the incidence rate of CAUTI in cerebral infarction patients, with adjustment for patient risk factors for CAUTI and the severity of cerebral infarction using the Japanese DPC database. The results showed no significant difference in the incidence rate of CAUTI between the standard catheter group and antimicrobial catheter group. On stratified analysis, however, the incidence rate of CAUTI in diabetic patients on insulin during admission in the antimicrobial catheter group was significantly lower than that in diabetic patients on insulin in the standard catheter group.

Our study has three strengths. First, we analyzed a large sample from the Japanese DPC database, which included a cumulative total of 14 million hospital admissions for all conditions in 2 years. A systematic review in 2006 reported that antimicrobial catheters appeared to reduce asymptomatic bacteriuria during use of less than 30 days, but the results were limited by the small sample size of many of the included studies.^15^ Indeed, some randomized trials of the effectiveness of silver-coated catheters conducted prior to 1995 examined only 90 to 171 patients. In contrast, our study enrolled about 27,000 patients from among approximately 300,000 cerebral infarction inpatients for analysis.

Second, we used patients’ clinical information in the analysis. A randomized crossover trial (*n* = 27,878) concluded that silver-alloy, hydrogel-coated catheters were significantly associated with lower incidence rates of CAUTI in a before-after analysis (*n* = 2,778), and showed that the introduction of antimicrobial catheters led to a 57% decrease in incidence rates after 1995.^16^^,^^17^ These studies were conducted in a single hospital or in several intensive care units, but the analyses did not consider patient background, such baseline disease, comorbidities, and complications, as well as treatment received by the patient, such as surgery, type of drug, and rehabilitation. We analyzed the effectiveness of antimicrobial catheters in preventing CAUTI with adjustment for these factors. In fact, the present study found that these patient background factors were consistent with the risk and preventive factors reported in previous studies.^4^^,^^18^^–^^24^ Additionally, we limited the analysis to patients with acute cerebral infarction.

Third, we employed a multilevel model for analyzing multicenter data. A multicenter randomized controlled trial in 2012 showed that the use of catheters impregnated with nitrofural was significantly associated with a lower incidence rate of CAUTI.^8^ Another retrospective before-after study of urinary catheters in patients whose urine culture tests were positive reported a significant reduction in CAUTI rates.^9^ These trials were conducted in several centers but did not consider the differences between hospitals. We used multilevel analyses to adjust for differences between hospitals and considered case numbers over a 2-year period.

We performed subgroup analyses, in which cases were stratified by risk factors for CAUTI, to verify the preventive effect of antimicrobial catheters in each group. The multivariate multi-level analysis showed no significant difference in CAUTI rates between the standard catheter group and antimicrobial catheter group. However, stratified analysis revealed that diabetic patients who were administered insulin during hospitalization in the antimicrobial catheter group had a significantly lower CAUTI rate than those in the standard catheter group. A previous study reported that glycosuria was associated with increased incidence of urinary tract infections and rapid growth of causative bacteria.^25^^,^^26^ Diabetic patients who were treated with insulin were considered to have glycosuria, so the increase in CAUTI rates in insulin-treated patients may be caused by elevated urinary glucose levels. The results indicated that antimicrobial catheters could have an effect in preventing CAUTI among patients with glycosuria.

We consider that our results from the Japanese DPC database were comparable with those of these previous studies. The CAUTI incidence rate in the antimicrobial catheter group was 2.1 per 1,000 patient days, consistent with the rate of 2.7 per 1,000 patient days in a previous study.^16^

Several limitations of this study should be acknowledged. First, we used the discharge diagnosis to determine CAUTI in this study. Previous studies employed the Symptomatic Urinary Tract Infections criteria of the National Healthcare Safety Network (NHSN), which include duration of catheter use, patient symptoms, and results of urine examination.^2^^,^^9^^,^^27^ We were unable to use the NHSN criteria because the DPC database is a health insurance claims database and does not contain information on length of catheterization or laboratory examination data. However, the DPC database does include complications that affect the clinical course, and we therefore consider that our definition of CAUTI is similar to the Symptomatic Urinary Tract Infections criteria. Second, we did not differentiate between types of antimicrobial catheter because these were considered the same in the Japanese reimbursement system during the study period. Previous studies defined specific catheter types as an antimicrobial catheter or control catheter.^7^^–^^9^^,^^16^^,^^17^^,^^28^^–^^30^ These differences hampered comparison of our results with those of previous results. Third, although we used patient and hospital factors that might be possibly linked with the incidence of CATUI for adjustment, there was a possibility of residual confounding. In addition to the factors that have already been pointed out, such as the duration of catheter use, the result of urine examination or the antimicrobial mechanism of catheter, a meta-analysis reported that reminder systems that persuade doctors to remove unnecessary catheters were significantly associated with a lower CAUTI incidence rate.^5^ Further studies are needed to reveal the real preventive effect of the antimicrobial catheter. Furthermore, a relatively large number of cases was excluded because of a lack of background information, such as ADL.

In summary, we performed a multi-level logistic regression analysis using the Japanese DPC database to elucidate the effectiveness of antimicrobial catheters in the prevention of CAUTI in patients with acute cerebral infarction. The incidence rate of CAUTI in the antimicrobial catheter group was not significantly less than that of the standard catheter group. However, the results suggested that antimicrobial catheters did reduce CAUTI incidence in diabetic patients who are treated with insulin. Further studies are needed to reveal the effectiveness of antimicrobial catheters in the preventing CAUTI in diabetic patients.
